# A Review of Key Technologies in Gravity Matching Navigation

**DOI:** 10.3390/s26134208

**Published:** 2026-07-03

**Authors:** Jinqi Zhao, Zhaofa Zhou, Zhili Zhang

**Affiliations:** School of Missile Engineering, Rocket Force University of Engineering, Xi’an 710025, China; zhaojinqi0107@outlook.com (J.Z.); zzl202@hhu.edu.cn (Z.Z.)

**Keywords:** gravity matching navigation, inertial navigation, gravity reference map, suitable area evaluation, matching algorithm, integrated navigation, path planning

## Abstract

The passive nature of gravity matching navigation, along with its concealment and freedom from error accumulation over time, is essential for reducing inertial navigation system (INS) errors and enabling high-precision autonomous underwater positioning. The current paper provides a systematic review of major technologies in the field, including the development of underwater gravimeters, construction of gravity reference maps, suitable area selection, optimization of matching algorithms, gravity–inertial integrated navigation, and path planning. We discuss hardware developments, including classical sensors, gradiometers, and quantum sensors, as well as methodological concepts such as multi-source sensor data fusion, intelligent area selection, algorithm optimizations, connections between multiple filters, and intelligent trajectory design. Despite a relatively well-developed technical infrastructure, several bottlenecks remain, including the low engineering maturity of high-end hardware, poor algorithmic performance under extreme conditions, over-reliance on simulation, and weak module integration. Future research should focus on hardware miniaturization, cross-domain intelligent adaptive algorithms, multi-condition real-world validation, and the transition from loosely coupled to tightly coupled architectures to achieve improved accuracy and robustness.

## 1. Research Background and Significance

The ocean is a strategic space in which sustainable human development has become a priority, and it also imposes stringent requirements on underwater navigation technologies in terms of resource exploitation and rights protection. Satellite navigation is vulnerable to loss of service, whereas acoustic navigation risks target exposure and is susceptible to marine environmental noise. Although INS are passive and covert, their navigation errors grow continuously over time, leading to rapid degradation of long-endurance navigation accuracy. Gravity matching navigation has emerged as a viable alternative. Its fundamental benefits—passivity, concealment and freedom from the integration of inertial bias over time—ensure that it becomes the main solution to the problem of INS error-correction and the ability to operate in an autonomous underwater environment with high precision and a long duration.

Gravity matching navigation operates by measuring marine gravity anomalies in real time using onboard gravimeters. This measured information is then compared with a pre-stored high-precision gravity reference map to estimate the actual position and correct INS cumulative errors. This technology can be traced back to the 1970s when the U.S. Navy developed it as part of its Trident submarine-launched missile navigation optimization efforts. The U.S. Navy universal gravity module (UGM) reduced latitude and longitude errors of the INS by 90 percent. Thereafter, the U.S. military implemented gravity field information compensation technology to provide high-precision underwater navigation of more than 90 days. In the 1990s, China began similar research efforts, and various universities have since made progress in algorithm simulation and principle validation. Nevertheless, there are still gaps in the hardware itself, reference map development, and even matching strength between China and the U.S./Russia.

**Figure 1:**
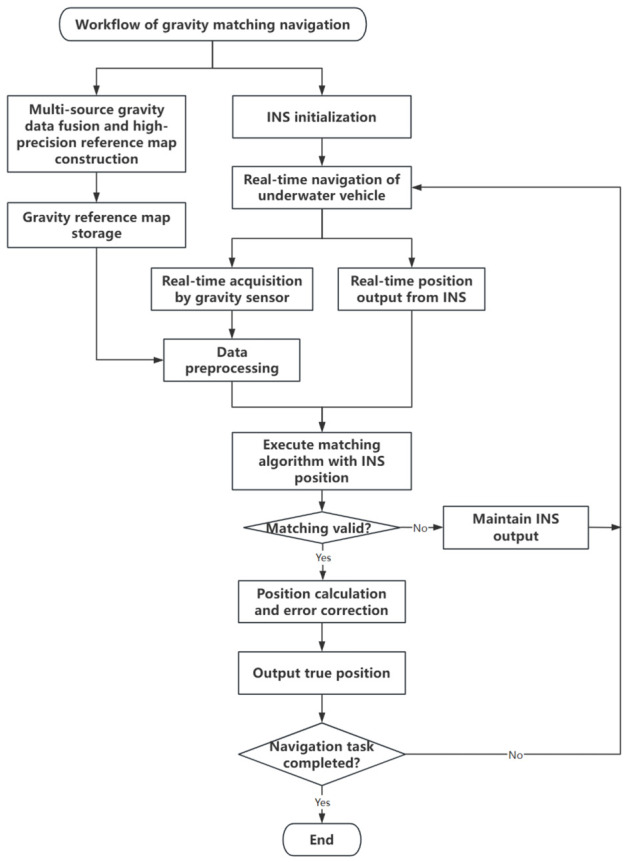
Workflow of gravity matching navigation.

The current practice in gravity matching navigation involves ingesting multi-source data and applying interpolation reconstruction to generate high-precision gravity reference maps. Then, suitable navigation areas are selected based on the features of the gravity fields. During navigation, the underwater craft obtains real-time gravity data and pre-processes it, combines it with the original INS position data, and compares it with reference map items using matching algorithms. After that, it finds the best true position and corrects the INS cumulative error, enabling iterative navigation. The exact steps are shown in Figure 1.

Unlike previous reviews that focus on single aspects such as matching algorithms or suitability area analysis, this review provides a systematic synthesis of six core technical directions—underwater gravimeter development, gravity reference map construction and data processing, matching area selection and evaluation, matching algorithm optimization, gravity–inertial integrated navigation, and path planning with track optimization—from the perspective of system integration and collaborative optimization. Particular emphasis is placed on analyzing technical bottlenecks, weak synergy among technical modules, and the future trend of transitioning from loosely coupled to tightly coupled architectures. This review aims to provide researchers with a holistic perspective to facilitate the transition of gravity matching navigation technology from isolated studies to system integration and from simulation-based validation to practical engineering applications.

## 2. Research Status of Key Technologies for Gravity Matching Navigation

### 2.1. Underwater Navigation Technologies

A comparison of underwater navigation technologies is shown in Table 1. Gravity-aided navigation has become the preferred choice due to its alignment with requirements for concealment, autonomy, and long-duration operation. However, the realization of these advantages requires underwater gravimeters to sense gravity features in real time and matching algorithms to estimate the true position for correcting INS errors.

**Figure 2:**
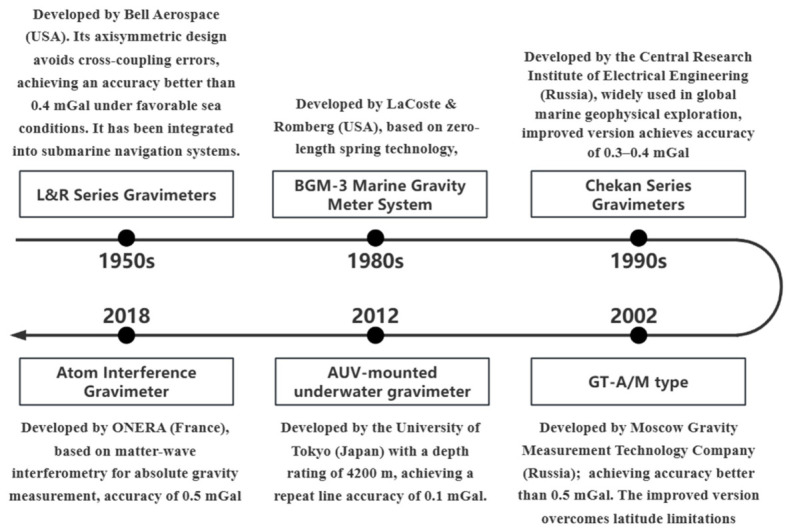
Development timeline of foreign gravimeters.

**Figure 3:**
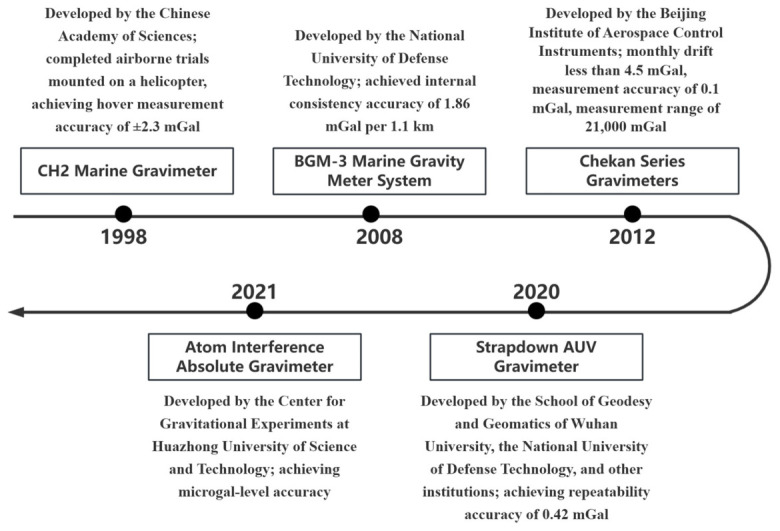
Development timeline of domestic gravimeters in China.

Global underwater gravimeter development is characterized by U.S. and Russia dominance with parallel progress in multiple countries, as illustrated in Figure 2. The U.S. and Russia began early and have adopted highly systematic approaches. Since the 1950s, the U.S. has developed the L&R series, BGM-3, and other marine/aviation gravimeters, while continuously advancing gravity gradiometer technology. GSS and subsequent UGM modules have been deployed on submarines, significantly improving INS accuracy [1,2]. Russia has introduced products such as the GT-2A/M series, with the improved GT-2AQ capable of measurements at geographic poles, demonstrating leading stability [1]. Japan has achieved a repeat track accuracy of 0.1 mGal using autonomous underwater vehicle (AUV) gravimeters [3], while France has attained measurement accuracy of 0.5 mGal with atomic interference gravimeters [4]. Multi-platform adaptation technology systems are gradually taking shape. Domestic independent research and development has been progressing since the 1990s, as shown in Figure 3. In recent years, through optimization of strapdown principles and error correction algorithms, China has achieved an underwater measurement accuracy of 0.42 mGal in AUV-mounted tests [5]. A prototype fountain-type atomic interference gravimeter at Huazhong University of Science and Technology demonstrated a sensitivity of up to 2.2 μGal/${Hz}^{\frac{1}{2}}$ in the laboratory [6]. For zero-length spring gravimeters, a two-layer temperature compensation algorithm (TLTCA) combining improved sparrow search algorithm (ISSA) and BP neural network achieved post-compensation standard deviation as low as 0.89 μGal under constant temperature conditions, while an improved alternating direction multiplier method (ADMM) for vibration compensation improved measurement accuracy from 3.60 mGal to 0.14 mGal [7,8]. Given the relative stability of current hardware capabilities, optimizing suitability analysis and matching algorithms at the system level is key to breaking through navigation accuracy bottlenecks [9].

Beyond hardware development, autonomous gravity measurement methods have also been explored as an alternative to global navigation satellite system (GNSS)-dependent approaches. For instance, an integrated SINS/odometer/altimeter system has been developed for land vehicles, achieving gravity anomaly accuracy of 0.49 mGal without relying on the GNSS. This technique shares fundamental principles with underwater gravity matching navigation, where GNSS signals are also unavailable, demonstrating the feasibility of fully autonomous gravity-aided positioning [10].

In brief, although China has taken some steps towards domestic equipment substitution, dynamic measurement, and quantum prototype design, substantial differences remain between China and the U.S./Russia in high-precision gravimeter design, extreme environmental suitability, and miniaturization of quantum devices. These hardware bottlenecks need to be addressed to enable future progress.

### 2.2. Gravity Reference Map Construction and Data Processing

**Figure 4:**
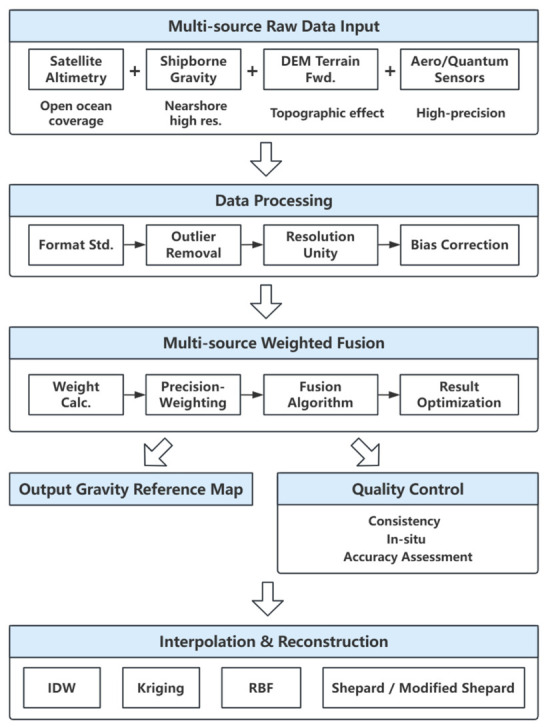
Workflow of gravity reference map construction.

The accuracy and resolution of the gravity reference map form the foundation for matching. Its construction has evolved from single-vessel surveys to a complete process involving multi-source data input, pre-processing, fusion, interpolation, and quality checks, as shown in Figure 4.

Shipborne and satellite altimetry data are inherently discrete, making interpolation reconstruction for improved reference map resolution and accuracy a central challenge. Traditional interpolation algorithms each have limitations. The Kriging approach provides optimal unbiased estimates by modeling the spatial correlation structure of the gravity field [11]:

(1) $${\hat{g}}_{Kriging}(x)=\sum_{i=1}^{N}\lambda_{i}(x)\cdot g_{i}$$

where ${\hat{g}}_{Kriging}(x)$ is the interpolated gravity anomaly at location x, $g_{i}$ is the measured gravity anomaly at the i-th control point, $\lambda_{i}(x)$ are the Kriging weights satisfying the unbiasedness condition $\sum_{i=1}^{N}\lambda_{i}=1$, and *N* is the number of surrounding control points. The weights are obtained by solving the linear system $\sum_{j=1}^{N}\lambda_{j}\gamma \left(x_{i},x_{j}\right)+\mu =\gamma \left(x_{i},x\right)$, where γ is the semi-variogram function and μ is the Lagrange multiplier.

Radial basis function (RBF) interpolation constructs the gravity field as a linear combination of radially symmetric basis functions centered at each control point [9]:

(2) $${\hat{g}}_{RBF}(x)=\sum_{i=1}^{N}\alpha_{i}\cdot \phi (∥x-x_{i}∥)$$

where ϕ(r) is an RBF, $\alpha_{i}$ are the weights determined by solving the linear system Φα=g (with $\Phi_{ij}=\phi (∥x_{i}-x_{j}∥)$), and $∥x-x_{i}∥$ is the Euclidean distance. Common choices for ϕ(r) include the multiquadric $\phi (r)=\sqrt{1+(\varepsilon r{)}^{2}}$, thin-plate spline $\phi (r)=r^{2}lnr$, and Gaussian $\phi (r)=e^{-(\varepsilon r{)}^{2}}$ functions, where ε is a shape parameter controlling smoothness.

The classical Shepard method estimates gravity anomalies as a distance-weighted average of surrounding control points [12]:

(3) $${\hat{g}}_{Shepard}(x)=\frac{\sum_{i=1}^{N}w_{i}(x)\cdot g_{i}}{\sum_{i=1}^{N}w_{i}(x)},w_{i}(x)=\frac{1}{d_{i}^{p}(x)}$$

where $d_{i}(x)=∥x-x_{i}∥$ is the Euclidean distance, and *p* is a positive power parameter (typically *p* = 2). The weighting function $w_{i}(x)$ decreases as distance increases, giving closer points higher influence. However, the classical Shepard method suffers from the “bullseye effect” around control points. To overcome this limitation, the Improved Shepard algorithm incorporates local quadratic corrections [13]:

(4) $${\hat{g}}_{\mathrm{ImpShepard}}(x)=\frac{\sum_{i=1}^{N}\left[w_{i}(x)\cdot Q_{i}(x)\right]}{\sum_{i=1}^{N}w_{i}(x)}$$

where $Q_{i}(x)=g_{i}+\nabla g_{i}^{T}(x-x_{i})+\frac{1}{2}(x-x_{i}{)}^{T}H_{i}(x-x_{i})$ is a local quadratic approximation of the gravity field around the i-th control point, with $\nabla g_{i}$ and $H_{i}$ representing the estimated gradient and Hessian, respectively. This enhancement significantly reduces the bullseye effect and improves interpolation accuracy.

In practice, the choice among Kriging, RBF, Shepard, and Improved Shepard involves a trade-off between accuracy, smoothness, and computational efficiency. As summarized in Table 2, Kriging provides rigorous error estimates but requires high-quality data and complex variogram modeling; RBF offers high accuracy for medium-scale complex fields but is sensitive to shape parameters; Improved Shepard balances global and local features at lower computational cost than Kriging, making it attractive for large-scale reference map construction. Integrated solutions, such as the Comprehensive Shepard method, combine multi-surface trend fitting with Shepard residual compensation, achieving superior performance in both accuracy and speed by avoiding the flaws of single algorithms. Despite these advances, studies have shown that interpolation errors remain pervasive; for example, the standard deviation of cubic function interpolation errors can still exceed 2.0 mGal [9]. Moreover, indiscriminately increasing the resolution of the reference map does not yield continuous accuracy improvements [12], indicating that algorithmic sophistication and data quality must advance together.

It is difficult for a single data source to balance spatial coverage and measurement accuracy: satellite altimetry offers good open-ocean coverage but low nearshore accuracy; shipborne data are rich in details but have limited coverage; digital elevation model (DEM) forward modeling can supplement terrain information but introduces model errors. Novel detection technologies provide new support for reference map construction. Multi-source data fusion is key to addressing this problem. By integrating global gravity field models, DEM forward modeling, and satellite altimetry data, discrete wavelet transform-based weighted fusion of high- and low-frequency components can achieve a balance between global contours and local details [15]. Techniques such as crossover adjustment [8] and collinear adjustment for satellite altimetry [16], as well as the right rectangular prism method for constructing gravity gradient maps, have also enriched data sources. Bian et al. systematically reviewed the technical contributions of gravity satellites such as CHAMP, GRACE, and GOCE, as well as the construction of the EGM2008 model, and noted that the effect of vertical deflection on platform INS positioning errors can exceed 2000 m [17]. This finding is consistent with our previous analysis, which demonstrated that the deflection of the vertical can cause nearly 3000 m of horizontal position error in INS [18]. Accurate modeling of the deflection of the vertical (DOV) is therefore essential for high-precision INS error compensation. A relative estimation method based on double alignment has been developed, where precise level alignment accuracy determines DOV estimation performance [19]. Furthermore, quantum gravity detection technology has demonstrated high-precision potential. By constructing a reference map based on cold atom interference gravimeters and seabed terrain forward modeling and combining it with the iterative closest contour point (ICCP) algorithm, the initial trajectory error can be reduced from approximately 4 km to 400 m [20].

The resolution of gravity measurements changes dynamically with speed, and asynchronous interaction with fixed-resolution databases degrades the correlation between matches. Also, measurement noise and outliers influence matching performance. To address this problem, Xu [21] proposed grid-sliding-window two-dimensional filtering technology that synchronizes measurements with database resolution in real time, reducing cumulative error to less than 0.8 nautical miles. The compound PPB algorithm, which integrates principal component analysis (PCA), particle swarm optimization (PSO), and back propagation neural network (BPNN), is applied to eliminate redundancy and improve data quality [22]. One technique that has improved the synergy of path planning and gravity matching is the generation of obstacle profiles based on the interpolation of terrain data along the path [23]. Beyond interpolation, gravity data post-processing often involves error separation and systematic adjustment. For example, empirical mode decomposition (EMD) has been successfully applied to airborne gravity data to separate dynamic errors, improving internal coincidence accuracy from 2.62 mGal to 0.75 mGal, with further enhancement to 0.28 mGal through repeated survey line adjustment [24].

Although considerable progress has been made, challenges remain in constructing a gravity reference map, including weak multi-source data fusion under extreme conditions, data bias correction, the inability of real-time dynamic map updating at high-speed maneuvers, and the lack of adaptive mechanisms to determine optimal resolution and interpolation parameters for different sea areas. In terms of validation, most interpolation algorithms have been evaluated using shipborne or satellite altimetry data, with some already incorporated into operational reference map production. However, real-time adaptive interpolation under dynamic conditions remains at the simulation stage, and few studies have validated their fusion methods under extreme marine environments—a gap that underscores the need for more field experiments to bridge the transition from simulation-based research to practical engineering applications.

### 2.3. Gravity Suitable Area Selection and Evaluation

The accuracy of gravity matching navigation depends strongly on the gravity field characteristics of the sea area. Regions with flat gravity field features are prone to ambiguity and mismatches. Therefore, accurate selection of suitable areas is critical to improve navigation success rates. Early studies often used single parameters such as standard deviation and roughness; however, practical applications have shown that single parameters cannot fully characterize gravity field properties and are prone to misjudgment. To address this, multi-feature fusion methods have been proposed, such as partitioning suitable areas using multi-attribute decision-making that integrates multiple feature parameters [25]:

(5) $$S(x)=\sum_{j=1}^{m}w_{j}\cdot f_{j}(x)$$

where S(x) is the comprehensive suitability score at location x, $f_{j}(x)$ denotes the *j*-th gravity field feature, $w_{j}\;$ is the corresponding weight, and m is the number of features. The weights can be determined objectively using principal component analysis (PCA) or factor analysis, which not only avoids subjective bias in single-parameter thresholds but also reduces feature redundancy and quantifies suitability using principal components or factor scores [26,27]. Experimental results indicate that the dual-feature criterion combining standard deviation and gradient change rate achieves the lowest mismatch rate [28].

Subsequent research has further optimized both feature extraction and dimensionality reduction techniques. In feature extraction, gray-level co-occurrence matrices and local binary patterns have been used to construct multi-dimensional texture and edge feature sets [29]. A Haar wavelet-based method extracts multi-directional high-frequency information, enabling direction-aware suitable area selection [30]. Feature importance ranking can screen optimal feature combinations and improve classification accuracy [31].

Traditional multi-feature fusion methods, relying on empirical thresholds, are subjective and lack robustness. To address this, fuzzy decision theory constructs a comprehensive evaluation index using membership matrices and fuzzy synthesis operations, avoiding the classification boundary distortion caused by hard thresholds [32]. Deep feature exploration algorithms, after Gaussian kernel-based dimensionality expansion and PCA reduction, use Gaussian mixture model (GMM) clustering to partition suitable areas, resulting in a significantly larger area of strongly suitable regions compared to traditional methods [33]. Furthermore, support vector machines (SVM) leverage their nonlinear classification capability to build a mapping between features and suitability, achieving good classification accuracy and recall rates [34].

The gravity field characteristics of special sea areas such as the Arctic and deep-sea smooth regions are unique, rendering traditional suitability evaluation methods inapplicable. For the Arctic, an adaptive threshold full-field extended extremum algorithm extracts multi-directional gradient features through convolution and adaptively adjusts thresholds based on positioning error feedback, achieving better screening performance than traditional algorithms [35]. For deep-sea smooth areas, a spatial-frequency multi-dimensional feature fusion algorithm transforms gravity sequences into the spatial-frequency domain and integrates multiple frequency- and spatial-domain features to construct similarity metrics, significantly improving matching success rates [36]. Methods based on gravity gradient tensors compute characteristic parameters such as trace and determinant. These methods can distinguish suitability even in smooth regions [37]. Related work has also expanded to cover global sea area suitability classification [38], independent confidence checks for matching [39], quantification of matching capability of areas (MCA) [40], and positioning accuracy measurement using the feature parameter Γ [41]. These developments have made the evaluation system more comprehensive.

To sum up, the suitable area selection technology has evolved into multi-feature integration, with specific applications in particular settings such as the Arctic and deep-sea level surfaces, as summarized in Table 3. However, there remain issues such as lack of data in special marine zones, restricted cross-domain extrapolation of intelligent models, poor coordination between selection and real-time matching, and inadequacy in automatic parameter adjustment. Moreover, many solutions lack real-world verification. Single-feature and multi-feature fusion methods have been validated using measured gravity data, whereas intelligent algorithms such as SVM, fuzzy decision, and deep feature exploration, as well as scenario-customized methods for the Arctic polar region and deep-sea areas, remain primarily simulation-based due to the scarcity of measured data in special sea areas. Future studies should focus on cross-sea intelligent modeling, dynamism in selection-matching synergy, parameter self-tuning, and engineering robustness through multi-condition real-world testing.

### 2.4. Gravity Matching Algorithm Optimization

**Figure 5:**
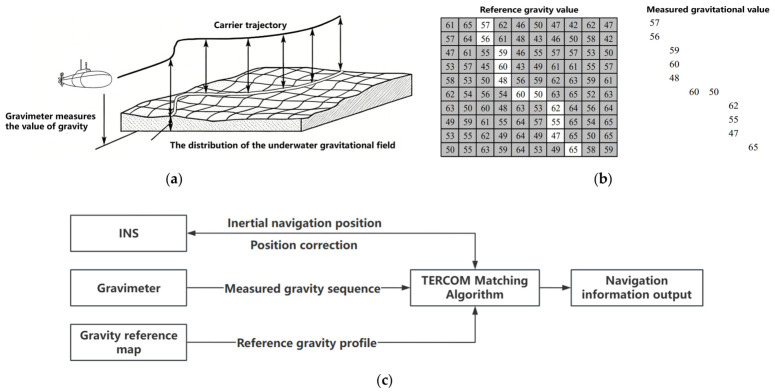
TERCOM Algorithm Principle Diagram. (**a**) Schematic of underwater gravity measurement, where a carrier traverses a gravity field while a gravimeter records gravity values along the trajectory; (**b**) Comparison between reference gravity values and measured gravity values used for matching; (**c**) TERCOM matching algorithm integrating INS, gravimeter, and gravity reference map for position correction.

**Figure 6:**
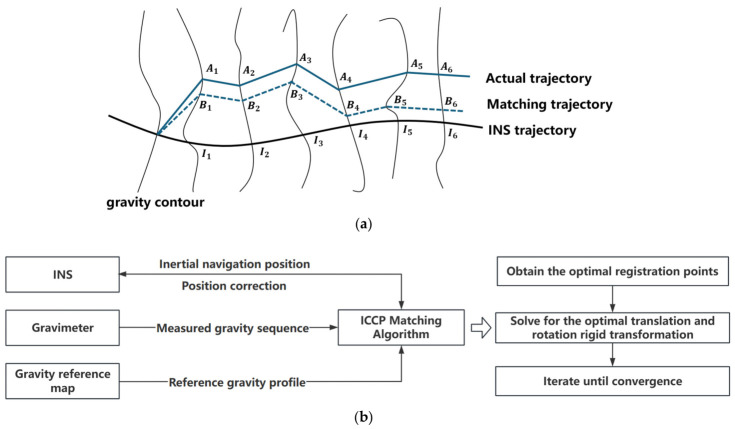
ICCP Algorithm Principle Diagram. (**a**) Trajectory comparison on gravity contour map among actual, matching, and INS trajectories with labeled waypoints; (**b**) Flowchart of the ICCP algorithm integrating INS, gravimeter, and gravity reference map to iteratively solve for optimal rigid transformation and correct navigation position.

**Figure 7:**
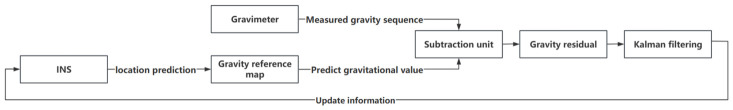
SITAN Algorithm Principle Diagram.

Gravity matching algorithms are central to achieving precise positioning. Early work was represented by three classical algorithms: Terrain contour matching (TERCOM), ICCP, and Sandia inertial terrain aided navigation (SITAN). TERCOM is based on correlation analysis, matching measured gravity sequences with reference map trajectory sequences. The algorithm searches over candidate positions to reduce the mean square difference (MSD) between the measured gravity profile and the corresponding reference gravity values:

(6) $$MSD(x,y)=\frac{1}{N}\sum_{k=1}^{N}{\left(g_{k}^{meas}-g_{k}^{ref}(x,y)\right)}^{2}$$

where N is the length of the gravity measurement sequence, $g_{k}^{meas}$ is the gravity anomaly measured at the k-th point along the trajectory, and $g_{k}^{ref}(x,y)$ is the reference gravity value at the candidate position (x, y) corresponding to the k-th point after accounting for INS-indicated movement. The estimated position is the candidate that reduces the MSD. ICCP originates from the ICP algorithm in image registration, achieving positioning through iterative approximation of contour trajectories. The core optimization problem is to find a rigid transformation that reduces the sum of the squared distances between the transformed INS trajectory points and their corresponding closest points on gravity contours:

(7) $$J(R,t)=\underset{R,t}{min}\sum_{k=1}^{N}{∥R\cdot p_{k}^{INS}+t-p_{k}^{contour}∥}^{2}$$

where $p_{k}^{INS}$ is the k-th INS-indicated position, $p_{k}^{contour}$ is the closest point on the gravity contour to the transformed INS point, R is a rotation matrix, and t is a translation vector. The solution is typically obtained through singular value decomposition. SITAN is based on an extended Kalman filter (EKF) framework, transforming matching localization into a state estimation problem. The system is described by the state equation and observation equation:

(8) $$x_{k+1}=F_{k}x_{k}+w_{k}$$

(9) $$z_{k}=h(x_{k})+v_{k}$$

where $x_{k}$ is the state vector, typically including position errors and other INS error states, $F_{k}$ is the state transition matrix, $w_{k}$ is process noise, $z_{k}$ is the observation, h(·) is the nonlinear observation function that maps states to predicted gravity anomalies, and $v_{k}$ is measurement noise. The EKF linearizes around the current estimate to perform recursive state update. To further elaborate the working mechanisms and implementation logic of the three classical gravity matching navigation methods, the basic principles and flowcharts of TERCOM, ICCP, and SITAN are analyzed below (Figure 5, Figure 6 and Figure 7). A comparison of their characteristics is presented in Table 4.

To balance positioning accuracy and real-time performance, a coarse-to-fine two-stage matching architecture has become the mainstream optimization approach, addressing the limitations of traditional single algorithms. This architecture adopts a pattern of coarse matching followed by fine positioning, effectively balancing computational cost and matching accuracy. Typical solutions include the following: using the MSD algorithm for rapid candidate trajectory screening in coarse matching, followed by ICCP-based fine correction, which improves positioning accuracy from kilometer-level to hundred-meter-level [44]; introducing affine transformation to rotate and scale the coarse matching results of TERCOM, compensating for trajectory deformation to further enhance accuracy [45]. The hierarchical neighborhood threshold search method also adopts a two-step strategy of coarse search and fine matching, significantly improving efficiency while maintaining accuracy [46]. Gao and Cai [47] proposed a cascaded MSD-EKF algorithm that uses MSD to mitigate large initial errors, incorporates matching results and gravity anomaly variations into the EKF observation equation, and integrates Doppler velocity log information, achieving stable matching under large initial errors. Xiao et al. [48] proposed a gravity lighthouse navigation method that breaks away from traditional sequence matching patterns. It uses multi-attribute decision-making to screen high-feature gravity areas as references and employs 2D image cross-correlation for area matching, achieving lower mismatch rates in smooth-feature sea areas. Although the coarse-to-fine matching architecture offers significant advantages, the setting of two-stage matching thresholds largely relies on experience and lacks adaptive mechanisms.

To address the tendency of ICCP to fall into local optima, a hybrid sparse ICCP joint robust method combines lp-norm-based coarse matching with classical ICCP fine matching, maintaining high accuracy despite measurement errors [49]. Such improvements retain the original algorithmic framework and have low engineering implementation difficulty, but their ability to strongly suppress nonlinear errors remains limited.

The SITAN algorithm primarily suffers from weak robustness, susceptibility to measurement noise interference, and significant linearization modeling bias. Current research addresses these issues by, on the one hand, using Huber robust equivalent weight estimation to dynamically adjust observation weights to suppress gross errors [43]; on the other hand, introducing a quaternion nonlinear error model combined with transformed cubature sampling filtering to compensate for the linearization deficiencies of traditional EKF, achieving significant accuracy improvements in real-ship tests [50]. To further overcome the linearization errors and measurement noise of SITAN, cubature Kalman filtering (CKF) combined with robust factors can effectively suppress observation gross errors [51]. In addition, point mass filtering (PMF) with simulated annealing-optimized resampling reduces the average matching error to within 500 m [52]. To adapt to different gravity field characteristics, a parallel fusion architecture based on standard deviation classification can adaptively switch between EKF, particle filtering, and Monte Carlo methods (MCM), keeping long-duration errors stable [53]. Unscented Kalman filtering (UKF) avoids truncation errors through unscented transformation, improving positioning accuracy by more than 30% compared to EKF [54]. Liu et al. transformed gravity sequences into the Hadamard domain, comprehensively considering numerical differences, trends, and spatial structures, and achieving high success rates even under harsh conditions [55]. Overall, filtering methods have clear theoretical advantages, but threshold design for multi-model switching and online computational burden remain major engineering bottlenecks.

To mitigate the limitations of TERCOM, such as the global exhaustive search and out of domain mismatch, related research has been performed on the parameter calibration, search domain optimization and adaptation to a special scenario. Through experiments, Wu et al. determined reasonable control ranges for sampling length, sampling interval, and systematic errors, providing guidance for engineering parameter configuration [56]. To address out of domain mismatches, approaches such as adaptive domain-center transfer, iterative double-cross domain-center transfer, cross-line adaptive domain matching, and alternating line small-domain matching have been continuously optimized. These methods improve the logic of global search and reduce computational redundancy [57,58,59,60]. Methods such as cyclic half-square domain iterative rematching, artificial bee colony algorithm with velocity limitation, and earth-axis projection dimensionality reduction matching are proposed to enhance the adaptability in complex sea areas including regions with dramatic gravity field changes and polar navigation [61,62,63]. Moreover, an enhanced vector matching algorithm incorporates phase constraint properties based on the short-term high-precision characteristics of INS [64]. The gravity anomaly difference full-line matching algorithm applies affine transformation for trajectory smoothing [65]. It uses TERCOM for coarse correction in non-suitable areas and PMF for fine matching in suitable areas. For mismatch problems, the priori recursive iterative least squares mismatching correction method (PRILSMC) method uses sliding-window recursive matching and iterative least squares to remove mismatched points [66]. Search domain optimization can reduce computation time and lower the probability of mismatches. However, a clear mapping between different methods and the sea area characteristics for which they are suitable has not yet been established.

Beyond the algorithms themselves, matching performance is also influenced by several connected factors. These include gravity database resolution, measurement fitness, sample size, and gravity field changes [67]. Algorithm design must be coordinated with gravity reference map characteristics. In recent years, machine learning has gradually been introduced into this field. Gao et al. proposed a K-nearest neighbor (KNN) matching algorithm [68]. It selects neighbors based on Euclidean distance and uses spatially weighted averaging to reduce the influence of outliers. This maintains a high success rate even under weak feature conditions. Zou et al. built trajectory direction and distance constraints using probabilistic neural networks. They improved accuracy and speed through two-step screening [69].

**Figure 8:**
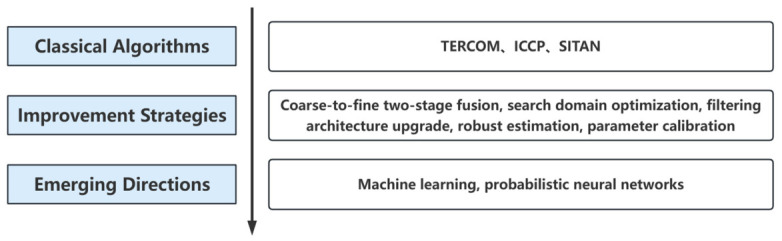
Evolution and classification of gravity matching algorithms.

In summary, matching algorithms have evolved into a system that integrates multiple methods and adapts to different situations. They have moved through classical, improved, and new stages, as illustrated in Figure 8. Current problems include low accuracy when switching between methods in complex gravity fields, difficult online self-tuning of key parameters, and the fact that most tests remain simulation based. Regarding validation status, the majority of matching algorithms, including most machine learning-based approaches such as K-nearest neighbor and probabilistic neural networks, as well as advanced filtering techniques like point mass filtering with simulated annealing, remain at the simulation stage. In contrast, algorithms that have seen real-world validation primarily belong to the classical category, including TERCOM, ICCP, SITAN, and their coarse-to-fine combinations, as well as certain robust variants of SITAN and transformed quaternion estimation methods. These have been validated using shipborne measurement data or reported in experimental studies. However, documented real-ship sea trials of gravity matching algorithms remain scarce in the open literature. This gap between simulation and real-world validation underscores the challenge of transitioning from academic research to practical engineering applications. Future breakthroughs require deeper integration of intelligent algorithms with filtering technologies and advanced parameter adaptive adjustment.

### 2.5. Gravity–Inertial Integrated Navigation

The quality of gravity–INS coupling directly determines navigation system performance. Early loosely coupled architectures only periodically output position correction information, making it difficult to suppress rapid divergence of INS errors. Moreover, they do not fully consider error propagation mechanisms, leading to a high risk of filter divergence.

To address the insufficient information interaction in loosely coupled architectures, a moving-window hierarchical model divides error levels based on INS circular error probable (CEP), constructs multi-layer parallel EKF filters and selects the optimal output using the minimum residual sum of squares criterion in a sliding window, achieving positioning errors ≤ 0.7 nautical miles over 24 h [70]. Federated Kalman filtering (FKF) processes gravity gradient and INS information through multiple sub-filters, uses adaptive information allocation factors to reduce measurement error interference, and combines improved adjusted spherical harmonic analysis (ASHA) to construct local gravity field models, improving long-duration accuracy and real-time performance [71]. The global state estimate in the federated filter is obtained by fusing the estimates from all sub-filters based on the information conservation principle [72]:

(10) $${\hat{x}}_{f}=P_{f}\sum_{i=1}^{n}P_{i}^{-1}{\hat{x}}_{i}$$

where ${\hat{x}}_{f}$ and $P_{f}$ are the fused state estimate and its covariance matrix, respectively; ${\hat{x}}_{i}$ and $P_{i}$ denote the local state estimate and covariance of the i-th sub-filter (e.g., one sub-filter for gravity gradient data, another for INS data); and *n* is the number of sub-filters. The inverse covariance weighting ensures optimal fusion in the minimum mean-square error sense. This federated architecture enhances both fault tolerance and computational efficiency, as the failure of any single sub-filter does not corrupt the global solution.

The error sources in integrated navigation systems are complex, and the unclear coupling relationships among them lead to poor suppression effectiveness. To address this, relevant studies have established an AR(1) model for gravity measurements and an interpolation error model, revealing through real-ship tests that the contributions of gravimeter and INS errors to matching errors are roughly equivalent [73]. Multi-source error collaborative suppression technology analyzes error propagation and constructs coupling models. It dynamically adjusts error weights using adaptive filtering. This improves matching success rates in nearshore areas [74]. Additionally, the MCA method based on conditional probability provides quantitative support for system parameter tuning [40]. MCM simulations evaluate error statistics by performing numerous random tests. This provides valuable information for error budgeting [75].

The gravity matching position estimates have a non-fixed output frequency which leaves conventional Kalman filtering vulnerable to divergence when sparsely populated. To address this, real-time correction algorithms detect discontinuous suitable segments along the entire trajectory, develop regression models relating gravity difference sequence variance to matching error, and dynamically determine fusion weights [76]. Multi-point comprehensive correction is a flexible INS correction method that uses SVM to assess the continuity of matching positions. EKF is applied for continuous positions, whereas multi-point comprehensive correction is used for sparse positions to estimate gyro drift and heading errors; however, this method can only estimate *Z*-axis gyro drift [77]. Sparse prediction using polynomial interpolation may limit the errors to reasonable levels during sparse conditions [78]. Additionally, three types of constraints—position, heading, and gravity—can maintain matching accuracy regardless of the magnitude of initial errors [79]. Navigation systems using simultaneous localization and mapping (SLAM) build gravity reference maps locally in real time, with the errors kept within 1.5 nautical miles in regions that do not have the pre-existing reference map [11].

In conclusion, gravity–INS coupling technology has evolved past the stage of loose coupling, and it is evident that filtering architectures have improved, error modeling has been enhanced, and sparse adaptations have been made. However, the following significant problems remain: partial knowledge of the nature of error coupling, inadequate dynamism of coupling to sparse sections of a journey, poor performance of multi-source error reduction in extreme situations, and an overall absence of real-ship testing. Regarding validation status, classical filtering architectures such as extended Kalman filter and federated Kalman filter have been validated through both simulation and sea trials, whereas sparse adaptation methods and multi-source error collaborative suppression techniques remain largely at the simulation stage with limited real-ship validation, particularly under extreme marine conditions. This validation gap reinforces the observation that while theoretical progress has been made, practical engineering validation lags significantly behind. Future studies should aim at quantitative analysis of error coupling, optimization of dynamic coupling algorithms, improvement of resilience to extreme conditions, and validation using multi-condition real-world data.

### 2.6. Path Planning and Trajectory Optimization

The main purpose of path planning and trajectory optimization is to generate trajectories that align with gravity characteristics while avoiding poorly suited regions and obstacles, thereby enhancing the overall matching success rate at the trajectory level. Early path planning typically focused on single objectives such as minimum distance or shortest time, without considering gravity suitability. This often resulted in planned paths crossing undesirable regions, leading to matching failure. Current research combines gravity field suitability characteristics, obstacle constraints, and vehicle maneuverability through three technological approaches: improved geometric search, swarm intelligence optimization, and three-dimensional multi-constraint planning, to achieve multi-objective trajectory optimization.

In terms of geometric search algorithm improvements, conventional search algorithms such as A* and rapidly-exploring random tree (RRT) have been adapted for gravity navigation applications. The multi-attribute decision-making approach combines various properties of gravity fields in order to divide appropriate regions. An improved version of A* with a new heuristic function and composite distance metric can reduce travel time and redundant turns [25]. Other studies have dynamically adjusted weights based on obstacle density and clearance rate, using a bidirectional Floyd algorithm to smooth paths, resulting in shorter paths and smoother turns [22]. In contrast to the grid-based search in A*, advanced RRT-based algorithms use a gravity suitability cost function to drive the growth of trees in order to cover more suitable areas when dealing with complex obstacles [64]. These methods provide significant path feasibility, yet the overall optimality is very sensitive to the cost function structure, and their dynamism to gravity field changes cannot be adequately addressed.

In swarm intelligence optimization, many bio-inspired intelligent algorithms have been introduced for trajectory planning. The particle swarm optimization–grey wolf optimizer (PSO-GWO) integrates the advantages of both methods. It uses PSO’s individual best memory to change the GWO update function. This balances path quality and convergence speed [80]. The A*-guided improved elite ant colony algorithm adds pre-planned paths into the ant colony state transition probability. It also uses elite strategies and smoothing factors. This significantly reduces the number of convergence iterations and path length [81]. Compared with geometric search, intelligent optimization algorithms have stronger global search ability. However, their convergence speed under multiple constraints remains low, and the results are sensitive to parameter choices.

In 3D and multi-constraint planning, the field is slowly moving from 2D plane planning to three-dimensional underwater navigation. A 3D along-path obstacle profiling method breaks planning into 2D plane planning and altitude planning. It makes obstacle profiles through pre-processing, coarse planning, and second-step optimization. This gives better efficiency and collision checking than older methods [23]. A layered planning framework handles global route planning through suitable areas at the top level and local obstacle avoidance and smoothing at the bottom level. This works well for complex seabed terrain [82]. 3D planning is more realistic for actual underwater navigation. However, the computational cost remains high, and most studies have not fully incorporated practical engineering constraints such as motion limits and turning restrictions.

Overall, path planning has evolved from single-objective range optimization to multi-objective development. It now includes gravity suitability, obstacle avoidance, smoothing, and maneuverability limits. However, intelligent algorithms have slow convergence speed under many constraints and do not respond well to changes in the gravity field. Also, most plans do not fully incorporate real maneuverability features. Regarding validation status, geometric search-based methods such as A* and RRT have been validated through simulation and some field tests, whereas swarm intelligence-based methods including particle swarm optimization–grey wolf optimizer and ant colony algorithms remain primarily at the simulation stage due to their higher computational complexity and sensitivity to parameter settings. This validation gap underscores the challenge of transitioning from theoretical research to practical underwater navigation applications. Future progress requires dynamic sensing and real-time adjustment methods, as well as the incorporation of maneuverability constraints into planning models.

## 3. Literature Review and Commentary

This analysis shows that gravity matching navigation has built a relatively complete technical framework. It covers hardware, data processing, algorithms, and planning. The U.S. and Russia still do better in high-end gravimeter engineering and new detection methods. However, China is catching up with new ideas in matching algorithms, dynamic measurement, and quantum prototypes.

However, the following significant bottlenecks persist:

1. Hardware: Insufficient engineering maturity and miniaturization of high-end equipment
2. Algorithms: Poor robustness and environmental adaptability in extreme marine conditions
3. Validation: Limited real-world marine measurements for most proposed schemes
4. System Integration: Weak dynamic coordination and adaptive coupling among sub-systems.

Addressing these issues requires future research to focus on the following:

1. Hardware Advancement: Closing existing hardware gaps
2. Error Mechanisms: Deepening the understanding of error coupling
3. Algorithm Development: Creating cross-domain intelligent adaptive algorithms
4. Validation: Strengthening multi-condition real-world validation.

Promising future directions include closed-loop feedback between suitable area selection and path planning, adaptive parameter setting, multi-heading suitability assessment, and directional map construction. These efforts can help transition technical components from loosely coupled to tightly coupled architectures, thereby making the system more effective and reliable in challenging environments.

## Figures and Tables

**Table 1:** Comparison of core characteristics of underwater navigation technologies.

Navigation Category	Advantages	Disadvantages	Position Accuracy	Engineering Maturity
Inertial Navigation	Strong autonomy, short-term accurate	Errors accumulate	0.1–1 n mile/24 h (Navigation-grade)	Operational application
Acoustic Navigation	High accuracy with baseline systems	Poor concealment, high cost, externally dependent	LBL: Sub-meter to meter-level, USBL: 0.1–1% of slant range	Operational application
Satellite Navigation	High accuracy	Water-blocked signals	Meter-level (on the surface)	Operational application
Terrain Navigation	Passive, strong concealment	Limited by flat terrain	Hectometer-level (in featured terrain)	Sea trial
Geomagnetic Navigation	Passive, strong concealment	Magnetic interference, map update needed	Sub-hectometer-level (in magnetic anomaly areas)	Primarily simulation, lake trial
Gravity Matching Navigation	Passive, concealable, broad coverage depth-unconstrained, interference-resistant	High hardware demand, poor dynamic adaptation, initial error sensitive	Hectometer-level (in gravity-favorable areas) Kilometer-level or higher (in weakly featured areas)	U.S./Russia: operational, China: simulation and lake trial

**Table 2:** Comparison of commonly used gravity interpolation algorithms.

Algorithm	Basic Principle	Advantages	Disadvantages	Interpolation Accuracy
Shepard Method	Inverse distance weighted average	Simple, low computational complexity, easy to implement	Bullseye effect, poor smoothing	Low
Kriging Method	Variogram-based optimal unbiased estimation	Rigorous, provides error estimate, smooth results	Complex modeling, high computational cost, requires prior knowledge	Highest (improved version: average error < 0.05 mGal) [14]
RBF Method	Fits points using RBF	Adapts to irregular data	Accuracy degrades under dramatic variations, parameter-sensitive, high cost for large samples	Relatively high
Improved Shepard Algorithm	Shepard with local quadratic correction	Balances global & local features, medium computational complexity	Requires parameter adjustment, unstable under extreme terrain	Higher than Shepard, close to Kriging
Comprehensive Shepard Algorithm	Trend fitting with Shepard compensation	Combines two algorithms, avoids single-algorithm flaws	Complex procedure, model-dependent multi-source compatibility	High, comparable to Kriging

**Table 3:** Evolution stages of suitable area selection technology for gravity matching navigation.

Stage	Core Idea	Key Methods	Engineering Maturity
Single-Feature Evaluation	Single indicators	Sliding-window smoothing, thresholding	High (early systems, simple to implement)
Multi-Feature Fusion	Integrate multiple features	PCA, factor analysis, buffer analysis	Medium (mature theory, gradual application)
Intelligent Algorithm	Machine learning replaces manual thresholds	SVM, fuzzy decision, ELM, RF, GMM	Medium–Low (primarily simulation)
Scenario Customization	Dynamic adaptation for special sea areas	Polar model, deep-sea spatial-frequency method	Low (insufficient research)

**Table 4:** Comparison of three classical matching algorithms.

Algorithm	TERCOM	ICCP	SITAN
Tolerance to initial error	Insensitive	Moderate (large errors prone to local optima)	Sensitive (prone to filter divergence)
Real-time performance	Poor (batch processing latency)	Moderate (time-consuming iteration)	Good (real-time filtering)
Computational complexity	Moderate (global search)	High (iterative coordinate transformation)	Moderate (filtering and linearization)
Resistance to environmental interference	Weak (requires prominent features)	Moderate (effective in specific correlation areas)	Linearization bias under dramatic gravity field variations
Positioning Accuracy	Coarse matching of <2000 m refined to <700 m with ICCP fine matching [42]	100–1700 m [42]	General SITAN achieves 3700–5600 m, refined to <2000 m with robust estimation [43]
Applicable scenarios	Coarse matching under large initial errors	Moderate errors, iterative fine matching	Requires small initial errors, real-time continuous correction

## Data Availability

No new data were created or analyzed in this study. Data sharing is not applicable to this article.
